# Self-Consolidating Lightweight Concrete Incorporating Limestone Powder and Fly Ash as Supplementary Cementing Material

**DOI:** 10.3390/ma12183050

**Published:** 2019-09-19

**Authors:** Muhammad I. Khan, Muhammad Usman, Syed A. Rizwan, Asad Hanif

**Affiliations:** 1 School of Civil and Environmental Engineering, National University of Science and Technology (NUST), Sector H-12, Islamabad, Pakistan; 2 National University of Computer & Emerging Sciences (NUCES-FAST), Faisal town, Lahore, Pakistan; 3 Institute of Applied Physics and Materials Engineering, University of Macau, Avenida da Universidade, Taipa, Macau, China

**Keywords:** self-consolidating, lightweight concrete, bloated aggregate, compressive strength, flexural behavior, fly ash

## Abstract

This paper assesses the mechanical and structural behavior of self-consolidating lightweight concrete (SCLWC) incorporating bloated shale aggregate (BSA). BSA was manufactured by expanding shale pellets of varying sizes by heating them up to a temperature of 1200 °C using natural gas as fuel in the rotary kiln. Fly ash (FA) and limestone powder (LSP) were used as supplementary cementing materials (10% replacement of cement, each for LSP and FA) for improved properties of the resulting concrete. The main parameters studied in this experimental study were compressive strength, elastic modulus, and microstructure. The fresh-state properties (Slump flow, V-funnel, J-Ring, and L-box) showed adequate rheological behavior of SCLWC in comparison with self-consolidating normal weight concrete (SCNWC). There was meager (2–4%) compressive strength reduction of SCLWC. Lightweight aggregate tended to shift concrete behavior from ductile to brittle, causing reduced strain capacity and flexural toughness. FA and LSP addition significantly improved the strength and microstructure at all ages. The study is encouraging for the structural use of lightweight concrete, which could reduce the overall construction cost.

## 1. Introduction

Although the use of lightweight concrete (LWC) dates back to 3000 B.C [1], the more significant work on lightweight concretes has been carried out in the last few decades [2,3,4,5,6,7,8]. The usage of lightweight concrete decreases the overall weight of concrete, resulting in reduced structural element dimensions [9]. This can result in the cost-effectiveness of structures such as long-span bridges and high-rise buildings. In addition to the lower weight, lightweight concrete shows better thermal resistance than ordinary concrete. Furthermore, due to its lightweight, porous structure, LWC exhibits excellent thermal conductivity, ease of placement, and better strength [10,11,12,13].

As per ACI 213R, structurally lightweight concrete has a unit weight ranging from 1350 to 1900 kg/m^3^, and a minimum compressive strength of 17 MPa [14,15,16,17,18]. The density of concrete made from natural aggregate, originating from hard rock, ranges from 2200 to 2600 kg/m^3^, as the aggregate occupies major volume in the concrete. Because of the porous structure of the lightweight aggregate (LWA), its absorption is very high, which leads to lower compressive strength. LWC has been produced by utilizing different sorts of lightweight aggregates (LWAs), such as expanded perlite [19,20,21,22,23,24], hollow glass beads [20,21,24,25], expanded clay [26], and expanded polystyrene beads [27,28,29,30,31,32].

However, the lightweight concrete mix design is different from conventional concrete. Using the conventional mix design will give rise to material segregation, as well as lower the strength by reducing the weight of aggregate. To avoid this problem, the mix design of self-consolidating concrete (SCC) was used for lightweight concrete [14]. The SCC is a type of concrete which deforms effectively and has high resistance to segregation as per the ACI committee 237R-07 [33]. The SCC flows to fill up the accessible space under its own weight and needs no compaction [34]. In SCC production, there is no typical mix-design procedure, and the production is conceivable with different ingredients. Each constituent and its properties may have a different impact on the self-compacting characteristic. Henceforth, a SCC mixture, prepared as per any given method, may not really show self-compacting properties. Trial mixes are essential for a final conclusion to be made [35,36].

Self-consolidating lightweight concrete (SCLWC) is a special type of concrete that has advantages of both LWC and self-consolidating concrete (SCC). Therefore, for a successful project, the use of lightweight concrete will provide an economical solution for various engineering applications. Using the mix design methodology based on SCC, it can be possible to design a SCLWC mixture with superior fresh and hardened properties [37,38,39,40]. Due to the characteristics of SCLWC, it is an attractive alternative to traditional concrete because of its high workability and a significant reduction in weight. In any case, there are limited investigations on the mechanical and microstructural properties of SCLWC [4,41]. The self-consolidating lightweight aggregate (SCLC) is a highly flowable concrete which has high segregation resistance while being light as well. It is, however, possible to achieve the desirable flowability of the concrete by adding superplasticizer or by increasing the paste content, but this may also cause the concrete to segregate.

The fresh concrete behavior depends essentially on the workability of the concrete. Thus, it is imperative for concrete to have satisfactory fresh state properties that will affect the behavior of hardened concrete, including strength and durability [4,41]. The mixture design of SCLWC does not follow the mix design of LWC or SCC precisely; be that as it may, the guidelines for both LWC and SCC still govern the SCLWC mix design [4]. In the literature, existing methodologies for the mixture design of SCC have focused on the fresh properties to attain the required flowability and self-compacting ability, instead of the compressive strength. Consequently, the strength requirement in SCLWC needs special consideration [42,43].

Research has been done on the self-consolidating lightweight concrete durability aspect, and it is concluded that SCLWC could achieve adequate flowability, better strength, and high durability [34,44]. Some of the researchers utilized normal and lightweight aggregate in various percentages to produce high-strength, self-compacting lightweight concrete (SCLC) [35,37]. It was seen that there was no significant difference in the design of SCNWC and SCLWC except the type of aggregate which was used. The change in the response of self-compacting cementitious systems occurs mainly due to supplementary cementing materials (SCMs) because of the porosity, particle size, shape, and morphology [45]. The use of SCMs like fly ash (FA) and limestone powder (LSP) is thought to give a better response due to the workability and fresh and hardened properties [46]. 

### Research Significance

Due to the lack of research in the field of SCLWC, especially using artificial aggregate such as bloated shale, further investigations are needed. This study was carried out to develop a lightweight self-compacting shale aggregate concrete from locally manufactured material (bloated/expanded shale). The effect of fly ash (FA) and limestone powder (LSP) on the fresh state properties, compressive strength, homogeneity, porosity, the microstructure of concrete and density of SCLWC, were also investigated to explore the possibility of using LWA as a full replacement of NWA (normal weight aggregates) for improving the various fresh and hardened characteristics of SCLWC. The cement was replaced by the SCMs, i.e., 10% of FA and 10% of LSP. Four formulations were selected. The first formulation was self-compacting normal weight concrete (SCNWC) using natural aggregate; the second formulation consisted of SCNWC and 10% of LSP and 10% FA as replacement of cement. The third formulation consisted of SCLWC, using bloated shale as coarse aggregate (SCLWC); the fourth formulation consisted of SCLWC plus 10% LSP and 10% FA as replacement of cement.

## 2. Materials and Methods 

### 2.1. Raw Materials

**Figure 1:**
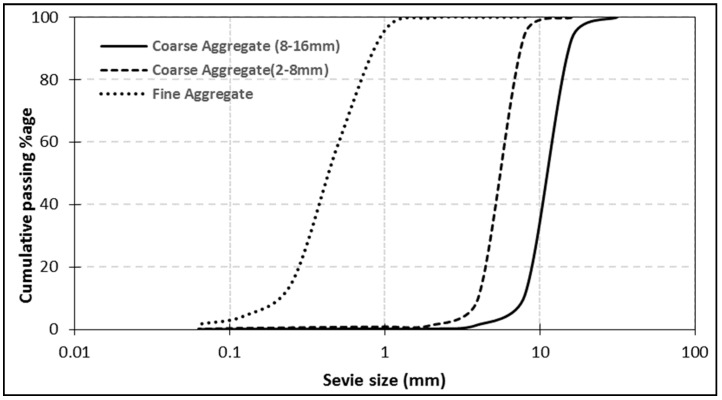
Sieve analysis of the coarse aggregate and fine aggregate.

**Figure 2:**
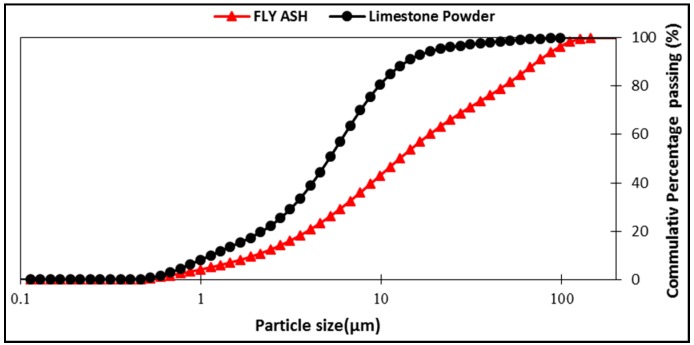
Particle size distribution of LSP and FA.

**Figure 3:**
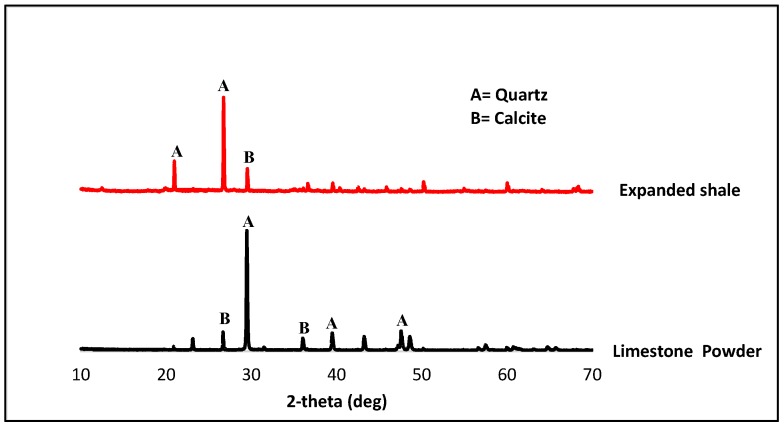
X-ray diffraction pattern of expanded shale and limestone powder.

The Ordinary Portland Cement (OPC) Type-I, conforming to ASTM C-150/C-150M-15, was selected as a binder for both SCNWC and SCLWC specimens. In this study, Glenium51, liquid superplasticizer (SP) conforming to ASTMC494, was used, while the fly ash was Class F with an apparent specific density of 2.68 g/cm^3^. The LSP used in this research was grayish in color and rich in CaCO_3_ (97.64%). It was made sure that both SCMs were free from lumps prior to mixing. The fine aggregate used was natural sand obtained from the Lawrencepur region, with a fine modulus of 2.24. Normal weight aggregate, comprising of crushed angular stone, was obtained from Margalla crush for the current research work. Coarse aggregates were used in two sizes—particle size of 2–8 mm and 8–16 mm. The maximum size for the coarse aggregate was 16 mm, conforming to ASTM C33, with a specific gravity of 2.47 (2–8 mm) and 2.44 (8–16 mm). Some of the physical properties obtained through lab tests are listed in Table 1. The test results of aggregate gradation performed on coarse aggregate are shown in Figure 1. The crushed limestone was used as coarse aggregate in SCNWC, while expanded shale was used as coarse aggregate in SCLWC. Some of the essential chemical and physical properties are given in Table 2 and Table 1. The sieve analysis performed on the fine aggregate and coarse aggregate is shown in Figure 1. The density, specific gravity, and water absorption of sand were determined as per ASTM C128. The particle size distribution (PSD) of FA and LSP was done by granulometric laser analysis, and it showed that the D50 of FA and LSP was 5.83 µm and 14.3 µm, respectively (Figure 2). The mineralogy properties of FA, LSP, and expanded shale has been studied by X-ray powder diffraction analysis (XRD) (Figure 3).

### 2.2. Mix Proportions and Mixing Methods

The objective of the study was to produce self-compacting yet lightweight concrete while using FA and LSP as SCM; therefore, corresponding attributes such as rheological behavior, unit weight, compressive strength, and microstructure of the resulting concrete was evaluated. Further, in order to ascertain the suitability of FA and LSP as SCM, corresponding material characterization techniques were employed. The SCC mix composition was designed by following the guidelines of EFNARC 2005 and ACI 237R-07. In this research, several trial concrete mixes using normal weight coarse aggregate and lightweight coarse aggregate with different superplasticizer dosages were prepared. From the numerous trial concrete mixes, suitable mixes satisfying the workability requirements for SCC (i.e., J-Ring test, L-Box, V-Funnel test, and Slump flow test) were selected. Four formulations for SCNWC and SCLWC were produced. For all the formulations, the water-cement ratio (w/c) was kept constant as 0.45. The mix proportion of various SCNWC and SCLWC mixes are shown in Table 3.

All of the materials were mixed in a pan mixer. First, coarse aggregates were put in the mixer, followed by sand and cement. One minute of dry mixing of the constituents at 180 rpm (slow rate) was done. Then, 50% of the water was added while mixing was continued at the same speed for another two minutes. The SP/viscosity enhancing agent (VEA) was then added along with the remaining water, and the mixing was further continued for three minutes at 360 rpm. The casting, curing, and testing was carried out as per EN 196-1. Concrete was cast into steel molds (three samples for each formulation for each testing age were cast). Cylinders of 150 mm diameter × 300 mm height and beams of 150 × 150 × 750 mm^3^ were cast for each formulation of SCNWC and SCLWC as per guidelines of (BS EFNARC 12390-1). A total of 48 cylinders and 12 beams were cast for all four formulations. The casted samples were demolded after 24 hours and placed in the curing tank, which contained water at the controlled room temperature. The samples were cured in water for a specified period of time before testing. 

### 2.3. Fresh Concrete Testing

#### 2.3.1. Density

The density/unit weight of fresh concrete was determined by measuring the weight of the container of known volume, fully filled with fresh concrete. The weight divided by the volume gives the density of fresh concrete.

#### 2.3.2. Air Content

The air content in freshly mixed self-compacting concrete was measured by following the standard ASTM C231 and EFNARC 12350-7 guidelines. The pressure method was used to determine the air content in concrete.

#### 2.3.3. Flow Tests for SCC

After the first mixing of SCC, the test was carried out in the following sequence:

- Slump flow
- V-Funnel
- L-Box
- J-Ring
- Sieve Stability Test (Segregation resistance)

### 2.4. Hardened Concrete Testing

#### 2.4.1. Density and Absorption

The density and water absorption of the concrete samples at the age of 28 days were determined as per the standard set forth by ASTM C642. The water absorption of the concrete specimen was measured as a percentage difference in the weight of concrete samples before and after the immersion in water at the age of 28 days.

#### 2.4.2. Compressive Strength Test

To assess the compressive strength of both SCNWC and SCLWC with the replacement of limestone powder and fly ash, a cylindrical specimen with a dimension of 150 mm × 300 mm cured at 3, 7, 14, and 28 days were tested in compression using a SHIMADZU universal testing machine (UTM) at a loading rate of 0.2 MPa per second as per ASTM C39.

#### 2.4.3. Microstructure

The microstructure was studied by scanning electron microscopy (SEM). For SEM, samples of both SCNWC and SCLWC after compression testing were selected to study the microstructure, morphology, and ITZ (interfacial transition zone). The sample preparation for both the tests were done in the laboratory by placing the sample in acetone (to stop hydration) for 24 hours, after testing of the cylinders after 28 days.

## 3. Results

### 3.1. Fresh State Concrete Properties

The results of the fresh properties of SCNWC and SCLWC are tabulated in Table 4. The fresh densities of SCNWC increase with the addition of mineral admixture i.e., limestone powder and fly ash. The densities of concretes containing lightweight aggregate are less than those containing normal weight coarse aggregate. It slightly increases with an increase in SCM but is still within the acceptable range. The SCLWC has a density 30% lower than SCNWC, while SCLWC + LSP + FA has 23% less density than the control mix. This is due to the porous and lightweight nature of the aggregate. The SP demand for all the four formulations with water to cement ratio (w/c) of 0.45, having a target flow of 70 ± 2, is also below the expectation. SCNWC requires 28.17% more SP than SCNWC + LSP + FA, while SCLWC requires 14.28% less SP than SCNWC, which is the control mix. This is due to the porous structure of the lightweight aggregate. SCLWC + LSP + FA requires 33% more SP than SCLWC owing to the high surface area of FA and LSP. 

All the values of slump flow and T50 are satisfactory, and within an acceptable range of SCNWC and SCLWC for a slump flow i.e., (650–800 mm) and the flow time (2–5 sec), respectively. Although it can be observed that slump flow increases when the lightweight aggregate is used, this may be due to its round shape and smooth surface. In the flow spread, SCNWC + FA + LSP has 3% more flow than SCNWC. This is because of the addition of FA and LSP, as well as higher SP content which enhanced the flow.

Similarly, all the values of V-Funnel flow test are within the acceptable limit, i.e., 6–12 sec. The V-Funnel flow time indicates viscosity, which depends upon the type of aggregate used. The V-Funnel flow time for SCNWC + LSP + FA is greater than the others, and is 7% higher than the control mix, due to the SCMs, while the V-Funnel flow for SCLWC is 6% less than control mix, due to the lightweight and porous nature of the aggregate. 

The L-Box test results are also within the permissible limits of SCNWC and SCLWC, i.e., 0.8–1.0. The lower value of H_2_/H_1_ shows the lesser ability of passing concrete through the L-Box. Also, the aggregate size effects the passing ability, as aggregate stuck between the steel rods reduces the passing ability. The replacement of normal aggregate with lightweight aggregate increases the passing ability and decreases the blocking tendency of the mix due to its round and regular shape and texture. Due to no interlocking of lightweight particles, the L-Box flow time also decreases with the use of lightweight aggregate. The control mix has a great height difference, which is probably due to a higher SP demand. SCLWC has 7% less value than SCNWC, which may be due to its porous nature and better flow.

The J-Ring flow and blocking index (Bj) for all four formulations are satisfactory and within the acceptable range of SCNWC and SCLWC. The J-Ring test measured the passing ability of concrete through steel rods. Literature suggests that the difference between slump flow and J-Ring values should be less than or equal to 50 mm for SCNWC and SCLWC for the better passing ability. It has been observed that J-Ring flow values increase with the use of lightweight aggregate, which may be due to better bonding and using a constant w/c ratio, i.e., 0.45. The J-Ring flow for SCLWC + LSP + FA is the highest and is 4.10% more than the control mix due to the round shape of the aggregate and the lightweight nature of the aggregate.

The segregation resistance/sieve stability test values are also reasonable and within the tolerable limit of SCNWC and SCLWC, i.e., (5–15%). All the SCNWC and SCLWC show good segregation resistance. The sieve stability for SCLWC is 10.5% less than the control mix. This is because of the round shape of aggregate, which increases the workability of concrete.

### 3.2. Compressive Strength

**Figure 4:**
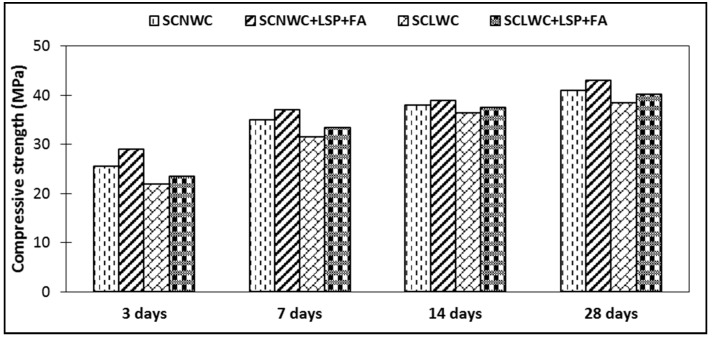
Compressive response of the concretes.

The compressive strength of SCLWC at 28 days was approximately the same as that of SCLWC. For typical lightweight concrete, the compressive strength decreases with a decrease in density. Figure 4 shows the change of compressive strength of SCNWC and SCLWC with respect to age. An average of three specimen results were taken as the representative strength. The data scatter was within 5% of the mean. 

The compressive strength of SCNWC and SCLWC increased from 25 to 41 MPa and 23 to 40 MPa at 3-day and 28-day age, respectively. The mix design for SCLWC and SCNWC targets the same compressive strength. One can get SCLWC of the same quality as normal SCC if the appropriate amount of SP, VMA, and lower water binder (w/b) is used. Due to the replacement of cement with FA and LSP, the compressive strength increases. This is due to the pozzolanic and filler effect of SCMs. The w/c ratio played a significant role in enhancing the 28-day compressive strength. The homogeneity and unit weight of SCLWC also depends upon the w/c ratio. Strength improvement with SCMs is more pronounced in concretes with lightweight aggregate.

### 3.3. Microstructure

**Figure 5:**
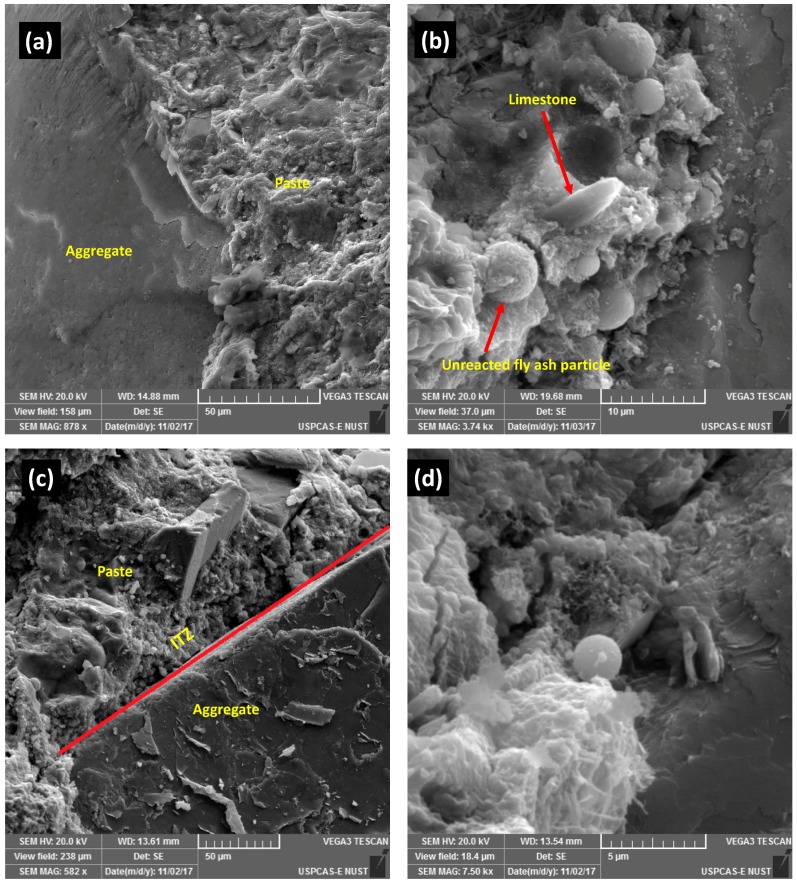
SEM of (**a**) SCNWC, (**b**) SCNWC + LSP + FA, (**c**) SCLWC, and (**d**) SCLWC + LSP + FA.

Figure 5 shows SEM images of SCNWC and SCLWC. The SEM images of the different concrete formulations were done to assess the porosity, ITZ, and microstructure qualitatively. The pozzolanic activity increases the hydration reaction and refines the microstructure of the concrete, which in turn enhances the concretes properties overall. FA and LSP incorporated specimens showed secondary hydrate formation as evident from the denser microstructure, which, thus, increased the compressive strength at later ages.

### 3.4. Summary of the Results

Based on the results and observations of an experimental investigation of the fresh and hardened properties of SCNWC and SCLWC, the following conclusions are drawn from this study:

- Proper pelletizing of clay was found to be essential for the efficient production of high-quality lightweight bloated shale aggregate.
- The most efficient part was the density of SCLWC, which was about 40% less than SCNWC. This is due to the lightweight nature of the aggregate. The bulk density of SCLWC was about 1700–1750 kg/m^3^, which is about 35–40% less than SCNWC.
- The 28-day compressive strength of SCLWC was 40 MPa, which was similar to SCNWC. The LWAC with fly ash as admixture increases the compressive strength of the concrete. Higher strength was achieved for a 10% cement replacement by fly ash. The optimum cement replacement by fly ash was around 10–20%. The 28-day compressive strength of SCLWC was quite comparable to SCNWC.
- The results show that modulus of elasticity of SCLWC was in an acceptable range. The E-value (modulus of elasticity) for the SCLWC was about 37% of that of SCNWC at the age of 28 days.
- Due to the acceptable workability with a medium water-to-cement ratio in SCLWC, there was no segregation or floating.
- Both SCMs (FA and LSP) enhanced the compressive strength up to a certain limit. The optimum replacement of FA and LSP would enhance the fresh and hardened response of the system.

## 4. Conclusions

A comprehensive and thorough study was carried out to examine the behavior of lightweight bloated shale aggregate on the performance of self-consolidating concrete. Based on the rheological properties, strength, and microstructural attributes, it was concluded that bloated shale could be used in concrete for producing self-compacting as well as lightweight concrete, while the subsequent properties can be further enhanced by the addition of mineral admixtures like FA and LSP.

- There was no significant (2–4%) reduction in the compressive strength of SCLWC. Lightweight aggregates tended to shift concrete behavior from ductile to a brittle.
- Incorporating the blend of FA and LSP enhanced the compressive strength of both SCNWC and SCLWC. This is because of pozzolanic and filler effects.
- The addition of lightweight aggregate significantly improved the workability of SCLWC. This is due to the round shape of LWA. The slump flow of SCLWC increased (1.39%) compared with SCNWC, while the flow time of SCLWC decreased (10%) compared with SCNWC.
- The addition of LWA reduced the density of SCLWC up to 35%. This reduction in density could reduce the overall cost of the structure because of deadload reduction.

The suitability of bloated shale aggregate for producing structurally lightweight self-compacting concrete and the positive effect of FA and LSP in improving the strength and microstructure are corroborated. This has significant applications in producing structures with greatly reduced deadloads, which would eventually reduce the overall cost of buildings due to reduced cross-sectional members and smaller foundations. However, due to the inherent brittleness of lightweight concrete, adequate techniques, like using short discontinuous fibers, should be adopted. 

## Figures and Tables

**Table 1:** Physical properties of fine and coarse aggregate.

Description	Coarse Aggregate (8–16) mm	Coarse Aggregate (2–8) mm	Fine Aggregate	Lightweight Aggregate
Max aggregate size (mm)	16	8	2	16
Fineness modulus	6.82	5.82	2.24	6.9
Specific gravity (SSD)	2.44	2.47	2.78	1.64
Water absorption (%)	0.7	0.5	1.62	5.02
Crushing (%)	21.63	26	-	39
Rodded bulk density (kg/m^3^)	1775	1597	1635	841.5

**Table 2:** Chemical composition of raw materials (wt %age). LSP, lime stone powder; OPC, Ordinary Portland Cement.

Description	SiO_2_	Al_2_O_3_	Fe_2_O_3_	CaO	MgO	K_2_O	Na_2_O	SO3	Cl
Fly Ash	55.32	0.26	6.54	6.78	1.22	2.39	0.19	1.14	-
LSP	8.64	0.84	0.82	46.76	1.65	0.10	0.02	0.11	-
OPC	20.51	5.25	3.39	61.53	2.33	0.77	0.31	2.84	0.01

**Table 3:** Concrete mix proportions (kg/m^3^). SCNWC, self-consolidating normal weight concrete; SCLWC, self-consolidating lightweight concrete.

Mix ID	Cement	Fly Ash	Limestone Powder	Water Content	Super Plasticizer	Coarse Aggregate (Normal Weight)		LWA	Sand	VEA
						(2–8 mm)	(8–16 mm)			
SCNWC	480	0	0	216	9.12	376	376	-	919	2.40
SCNWC + LSP + FA	384	48	48	216	8.06	376	376	-	919	1.92
SCLWC	480	0	0	216	11.00	-	-	504	919	2.40
SCLWC + LSP + FA	384	48	48	216	9.60	-	-	504	919	1.92

**Table 4:** Fresh state properties of concretes.

Mix ID	Density (kg/m^3^)	SP (%)	Slump Flow (mm)	Slump Flow Time (sec)	V-Funnel Flow Time (sec)	L-Box (H_2_/H_1_)	J-Ring Flow (mm)	J-Ring Blocking Step (mm)	Segregation Resistance (mm)
SCNWC	2338	1.4	720	2.2	9.60	0.86	700	6.0	9.73
SCNWC + LSP + FA	2342	1.8	740	2.5	10.28	0.83	720	7.0	9.51
SCLWC	1798	1.2	730	2.0	9.00	0.80	710	5.0	9.00
SCLWC + LSP + FA	1802	1.6	750	2.3	9.70	0.82	730	6.7	8.70
